# Managing Complex Pacemaker-Associated Endocarditis With Thromboembolism in Tbilisi, Georgia: A Case Report

**DOI:** 10.7759/cureus.80804

**Published:** 2025-03-18

**Authors:** Roin Rekvava, Tinatin Jomidava, Mariam Mamisashvili, Irakli Ninua, Ilia Khvichia, Natia Mirtskhulava, Irakli Gogokhia

**Affiliations:** 1 Electrophysiology, American Hospital Tbilisi, Tbilisi, GEO; 2 Pharmacology, European University, Tbilisi, GEO; 3 Microbiology, Tbilisi State Medical University, Tbilisi, GEO; 4 Infectious Disease, American Hospital Tbilisi, Tbilisi, GEO; 5 College of Medicine, Tbilisi State Medical University, Tbilisi, GEO; 6 Cardiology, American Hospital Tbilisi, Tbilisi, GEO; 7 Anesthesia and Critical Care, American Hospital Tbilisi, Tbilisi, GEO

**Keywords:** pacemaker infection, pacemaker lead extraction, pulmonary embol, staph aureus endocarditis, subacute bacterial endocarditis

## Abstract

Cardiac implantable electronic devices (CIEDs), including permanent pacemakers, implantable cardioverter-defibrillators (ICDs), and cardiac resynchronization therapy (CRT) devices, have become crucial in managing cardiac arrhythmias and heart failure. However, despite advancements in implantation techniques and prophylactic measures, CIED-related infections, including infective endocarditis (IE), remain a significant clinical challenge. These infections contribute to considerable morbidity and mortality, often requiring prolonged hospitalization, complex interventions, and significant healthcare costs. CIED-IE is particularly concerning due to its association with systemic complications, including septic embolization, which increases the risk of adverse outcomes.

We report the case of a 77-year-old male with a history of permanent pacemaker implantation who developed pacemaker-associated infective endocarditis complicated by septic pulmonary embolism. The patient experienced recurrent febrile episodes over a year and was intermittently treated with antibiotics without a definitive diagnosis. He later presented with fever, dyspnea, and generalized fatigue, prompting further investigation.

Transesophageal echocardiography (TEE) revealed vegetation measuring 1.39 × 2.75 cm on the pacemaker lead, and pulmonary CT confirmed bilateral septic emboli, indicative of septic embolization. Two sets of blood cultures were positive for methicillin-sensitive *Staphylococcus aureus* (MSSA) within 12 hours. Given the high risk of complications, a multidisciplinary team, including cardiologists, infectious disease specialists, and cardiothoracic surgeons, assessed embolic risks, infection control, and pacing needs. Broad-spectrum intravenous antibiotics and anticoagulation therapy were initiated. Despite medical management, the persistent infection and embolic risk necessitated transvenous lead extraction (TLE), which was performed under general anesthesia. A temporary pacing lead was inserted due to the patient’s pacemaker dependence. The procedure was successfully performed without complications, and no residual vegetations were observed on follow-up imaging.

## Introduction

Cardiac implantable electronic devices, such as permanent pacemakers, ICDs, and CRT devices, play a pivotal role in managing cardiovascular diseases [1,2]. Over the past few decades, the indications for and use of CIEDs have expanded significantly, with more than one million devices implanted annually worldwide, including over 400,000 in the United States [3,4]. These devices have been instrumental in reducing mortality associated with arrhythmias, cardiomyopathies, and ischemic heart diseases [2]. However, the widespread adoption of CIEDs has been accompanied by a steep rise in device-related infections (DRIs), particularly in patients with more complex devices, such as CRT, and those with advanced age and comorbidities [4,5].

CIED-related infections (CIED-IE) are among the most serious complications of device implantation, with an incidence of 0.6% to 3.4% depending on the population and device type [1,4]. The incidence varies based on multiple factors. Patients with CRT devices and ICDs have a higher risk compared to standard pacemakers due to increased lead complexity and longer procedural times; their patient-specific risk factors include older age, diabetes, chronic kidney disease, and prior CIED infections [1,4].

These infections can range from localized pocket infections to systemic infective endocarditis, which accounts for 10% to 23% of CIED infections and carries significant morbidity and mortality, with in-hospital death rates as high as 30% [3-5]. Infections are often associated with either direct contamination during device implantation or hematogenous seeding from distant bacteremia, leading to lead vegetation and systemic complications [3,5]. Biofilm formation on devices is a major potential challenge in eradicating CIED-IE, which justifies the necessity for complete device removal.

The management of CIED-IE requires a multidisciplinary approach, with early diagnosis and complete device removal being critical to achieving optimal outcomes [5,6]. Transvenous lead extraction is the gold standard for removing infected hardware, with infection resolution achieved through prolonged antimicrobial therapy targeted to culture results [2,7]. However, reimplantation strategies remain challenging, particularly in pacemaker-dependent patients or those with high arrhythmic risk, due to the potential for reinfection and gaps in international guidelines regarding timing, site, and device selection [2,4,6].

Advances in imaging modalities, such as three-dimensional echocardiography, positron emission tomography/computed tomography (PET/CT), and cardiac tomography, have improved diagnostic accuracy and characterization of CIED-related infections [7,8]. However, gaps persist in clinical practice, with many cases underreported or suboptimally managed [4,5]. This report highlights the successful management of a 77-year-old patient who endured a prolonged and debilitating course of pacemaker-associated infective endocarditis, culminating in life-threatening thromboembolic complications.

## Case presentation

A 77-year-old male presented to the emergency department on October 24, 2024, with complaints of fever, chills, dyspnea, and easy fatigability. His past medical history included pacemaker implantation in 2016 with complete atrioventricular block (AVB) and subsequent revision of the surgical site in 2018. Over the past year, he reported recurrent febrile episodes that were intermittently treated with antibiotics, although no definitive source of infection was identified.

As his condition worsened, a transesophageal echocardiogram performed on September 30, 2024, revealed thrombotic masses [1] on the pacemaker lead, leading to anticoagulation therapy with rivaroxaban, later switched to warfarin. Despite treatment, the patient’s symptoms worsened, prompting hospitalization.

Upon presentation, the patient was febrile at 39°C, tachypneic with a respiratory rate of 24 breaths per minute, and oxygen saturation of 90% on room air. Physical examination was significant for diminished breath sounds bilaterally with crepitus in the mid-lung fields and muffled heart sounds. Hemodynamic parameters were stable, with a blood pressure of 95/60 mmHg and a heart rate of 120 bpm.

Initial laboratory results showed leukocytosis (WBC 18.2 × 10⁹/L), anemia (hemoglobin 11.5 g/dL), elevated CRP (165.68 mg/L), and high D-dimer levels (1580 ng/mL) (Table 1).

**Table 1:** Laboratory results during hospitalization WBC: White blood count, RBC: Red blood count, CRP: C-reactive protein

Test	Units	Normal Range	Day 1	Day 4	Day 13
WBC	10^9/L	4.00 - 9.00	18.2	11.2	10.8
RBC	10^12/L	4.40 - 5.80	4.0	4.1	3.9
Hemoglobin	g/dL	13.50 - 18.00	11.5	13.0	12.0
Neutrophils	10^9/L	2.00 - 6.80	11.50	8.1	7.2
CRP	mg/L	<5	165.68	56.2	25
D-Dimer	ng/mL	<500	1580.0	450.0	400
Procalcitonin	ng/mL	<0.5	0.546	0.019	0.011
Lactate	Mmol/L	0.9-1.7	1.2	1.4	1.36
pH	-	7.31-7.43	7.4	7.36	7.33
Vancomycin Concentration	ng/ml	10-20	-	20.1	27.4

A thorough history revealed escalating shortness of breath over the preceding month, culminating in the current acute presentation. The patient denied a history of diabetes or asthma.

**Figure 1:**
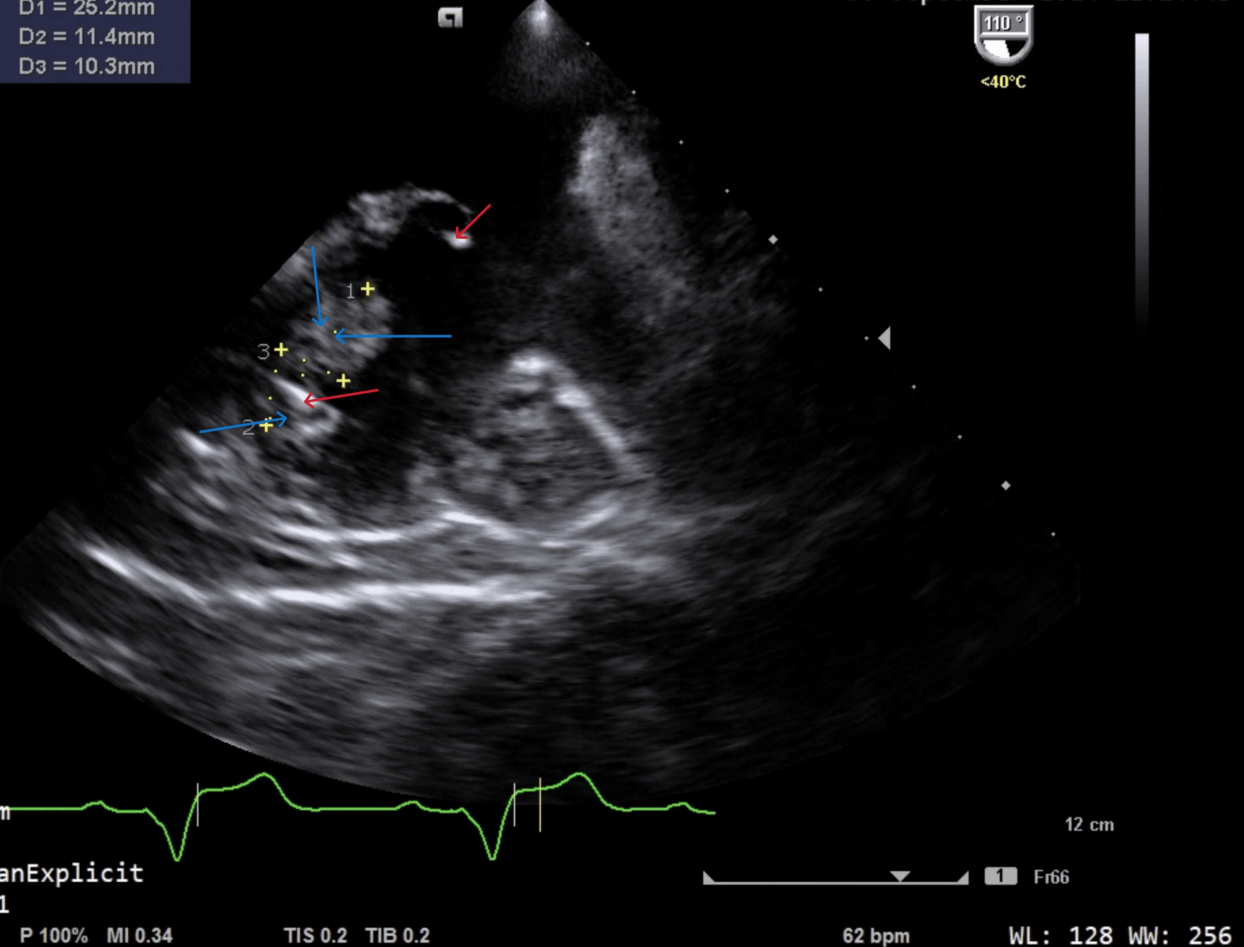
Initial echocardiography findings. Multiple hypermobile vegetations visualized on the pacemaker lead, prolapsing from the right atrium into the right ventricle. Red arrows: pacemaker leads, Blue arrows: infected masses

Initial echocardiography findings: The left ventricle showed normal size and systolic function with an ejection fraction of 58% and no regional wall motion abnormalities. The left atrium was mildly dilated, and diastolic function indicated impaired relaxation. Valvular findings included mild mitral regurgitation, mild aortic regurgitation, and moderate to severe tricuspid regurgitation. Multiple hypermobile vegetations (1.39 × 2.75 cm) were visualized on the pacemaker lead, prolapsing from the right atrium into the right ventricle (Figure 1). The right atrium was moderately dilated, the right ventricle functioned normally (TAPSE 23 mm), and pulmonary artery systolic pressure was significantly elevated at 62 mmHg. The inferior vena cava was 2.4 cm with >50% collapsibility, and no pericardial or pleural abnormalities were observed.

**Figure 2:**
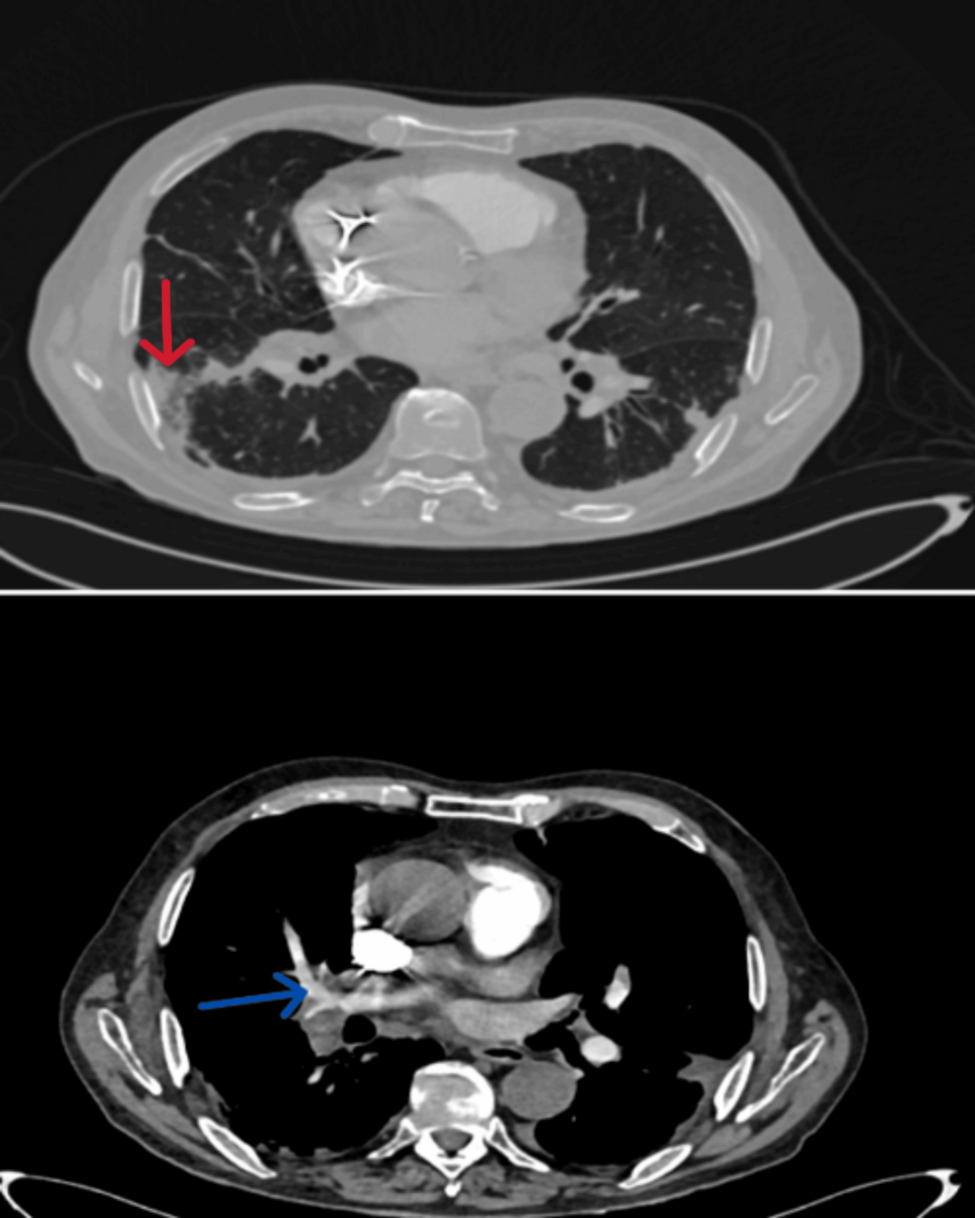
CT scan of bilateral pulmonary emboli with thrombotic masses in the right pulmonary artery and lower lobe branches. Consolidated focal infiltrates in the right lower and left upper lobes. Red arrows: Consolidated focal infiltrates, Blue arrows: Bilateral pulmonary emboli with thrombotic masses in the right pulmonary artery and lower lobe branches

Initial pulmonary CT angiography revealed no thoracic deformities or soft tissue pathology. Pacemaker leads were visualized in the superior vena cava and right atrium/ventricle. Bilateral pulmonary emboli were noted, with thrombotic masses in the right pulmonary artery and lower lobe branches (Figure 2). Consolidated focal infiltrates in the right lower and left upper lobes suggested infarct pneumonia. Mild fibrosis, small bilateral pleural effusions, and adjacent atelectasis were observed. The ascending aorta measured 46 mm, with calcified atherosclerosis in the aortic arch. No enlarged mediastinal or supraclavicular lymph nodes were present.

The clinical findings strongly suggested pacemaker-associated infective endocarditis complicated by septic pulmonary embolism. Initiated broad-spectrum antibiotic therapy with intravenous ceftriaxone (2 g every 24 hours) and vancomycin (1 g every 12 hours). Anticoagulation therapy was continued with heparin infusion.

Given the size and mobility of the vegetation, along with the embolic complications, the case was discussed in a multidisciplinary team meeting involving cardiologists, cardiothoracic surgeons, and infectious disease specialists. It was decided to proceed with transvenous removal of the pacemaker lead to prevent further embolic or systemic complications.

**Figure 3:**
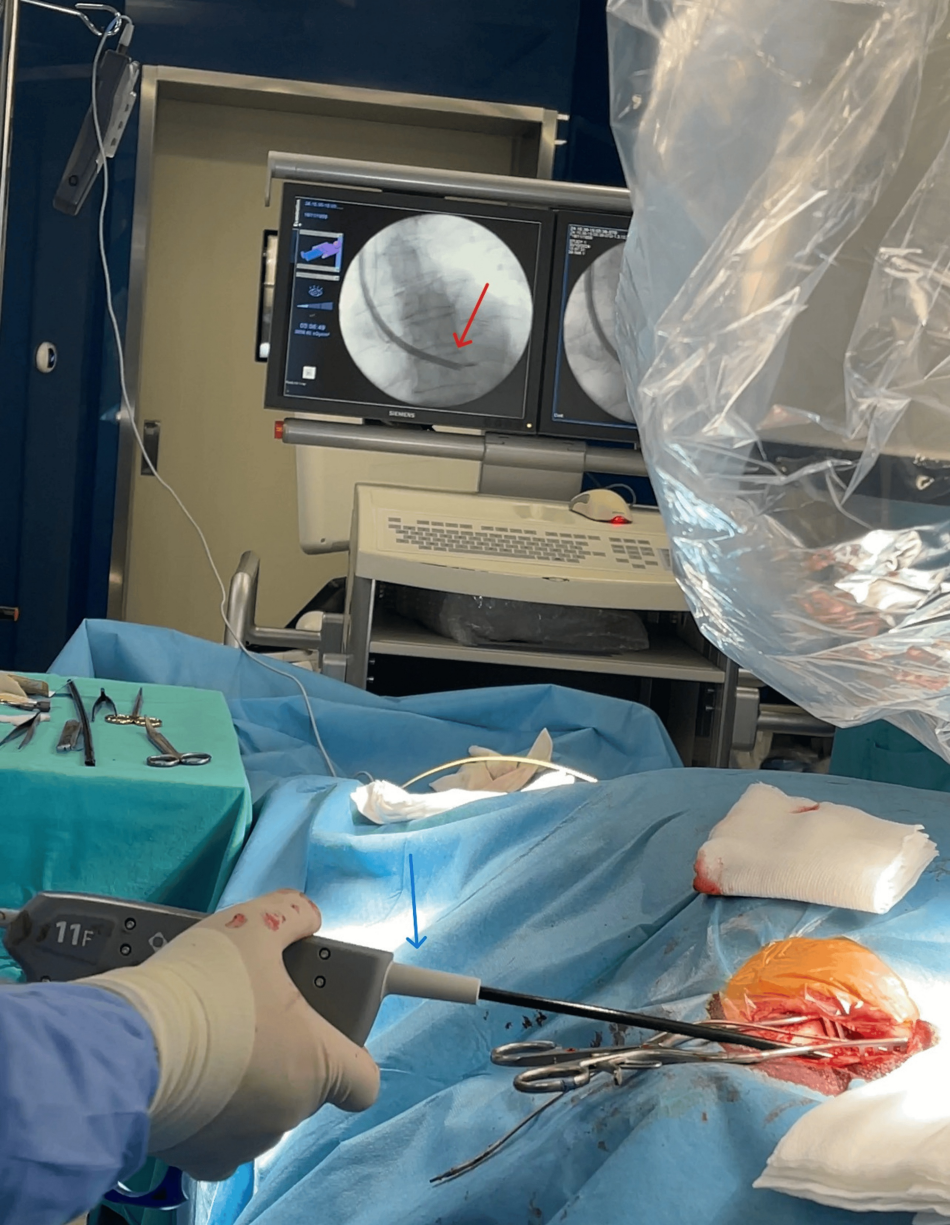
Lead extraction procedure Red arrow: Lead extraction procedure. A chest X-ray checks the position of implanted leads. Blue arrow: Lead extractor

On October 30, 2024, the patient underwent a transvenous pacemaker lead extraction under general anesthesia (Figure 3). Since the patient was fully pacemaker-dependent, a temporary pacing lead was inserted to ensure continuous cardiac rhythm management.

The procedure involved creating a 5 cm incision in the subpectoral (subclavian) region to access the pacemaker pocket. The pacemaker generator was carefully released, and the leads were detached. A locking stylet was introduced into the leads, and using the TightRail Mechanical Rotating Dilator Sheath (Philips, Amsterdam, Netherlands), a transvenous approach was employed to extract the ventricular lead first, followed by the atrial lead. The incision was closed with knot sutures after ensuring proper lead removal.

**Figure 4:**
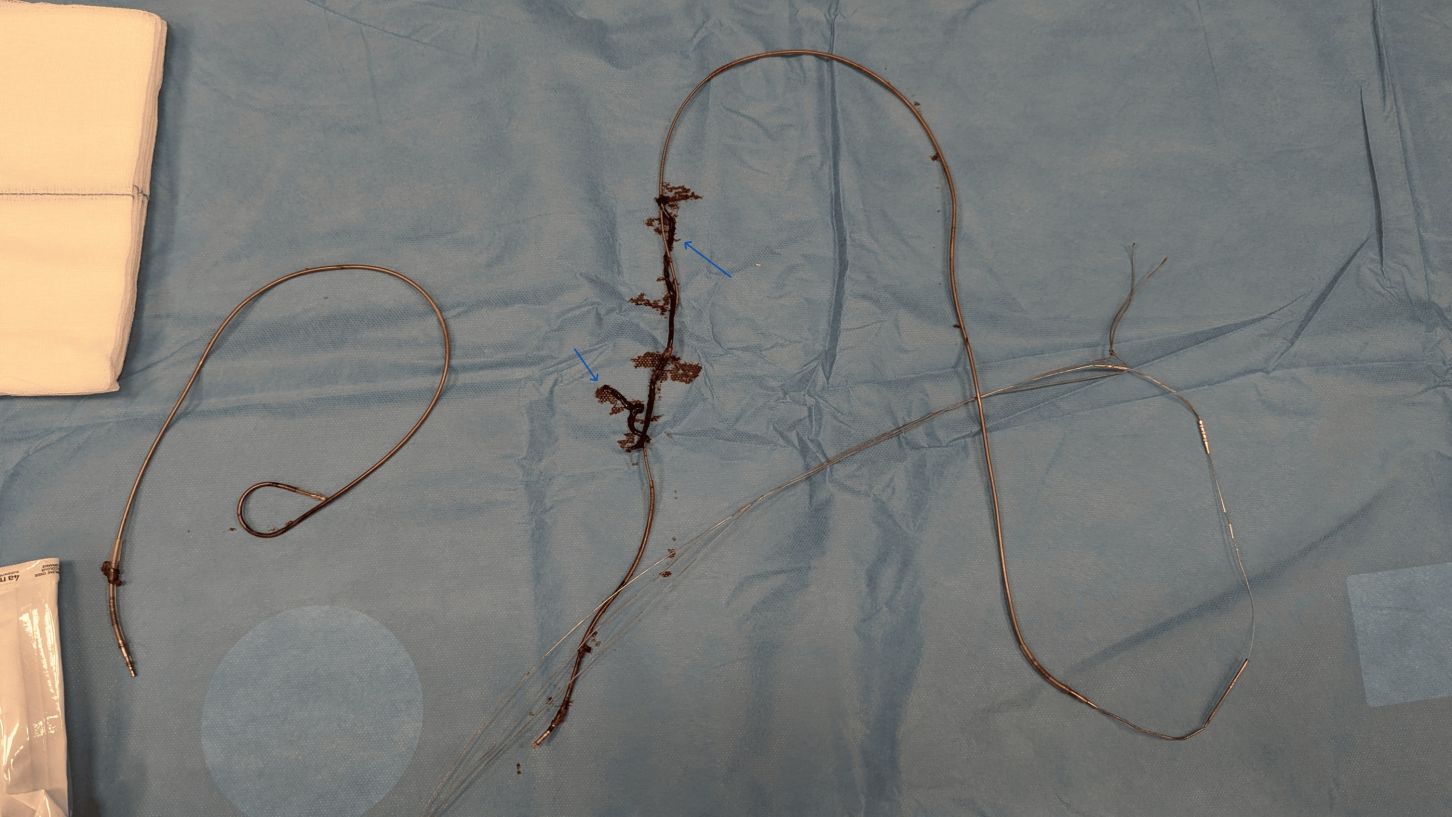
Lead extraction successfully performed, visible infected mass present Blue arrows: Infected masses

Under echocardiographic and fluoroscopic guidance, the transvenous extraction was performed successfully, and the leads were completely removed. The distal segment of the lead, which contained visible macroscopic material, was sent for bacteriological analysis (Figure 4).

Post-extraction, the integrity of the lead was carefully inspected to confirm no remnants remained in the patient. The operation was completed in 40 minutes without any complications.

Post-surgery, the patient showed marked clinical improvement: fever resolved within 48 hours, and respiratory symptoms significantly improved. Extracted mass culture revealed the same MSSA bacteria as in two previous blood specimens.

Repeat echocardiography on the second day of surgery revealed no residual vegetation and pulmonary CT imaging indicated partial resolution of thromboembolic defects. The patient was transitioned to oral anticoagulation therapy with close INR monitoring.

The patient was discharged 13 days after admission and five days after surgical intervention device implantation in the contralateral subclavian area [1] with the following recommendations: Continue oral anticoagulation with warfarin, with INR target 2.0-3.0; complete a six-week course of antibiotic therapy (ceftriaxone and vancomycin); schedule follow-up with cardiology and infectious disease specialists. The plan includes repeat imaging in 4-6 weeks to confirm the resolution of pulmonary embolism and assess cardiac function.

## Discussion

This case highlights the complexity and critical nature of managing pacemaker-associated infective endocarditis compounded by thromboembolic events. The rising prevalence of CIED-IE, attributed to increased device implantation rates and an aging population, underscores the importance of early detection and multidisciplinary management [3,9].

The incidence of CIED-IE varies between 0.5% and 2.2% annually, with mortality rates ranging from 10% to 30% [10,11]. The embolic complications seen in this patient are consistent with findings from several studies that report systemic embolization, particularly to the lungs, as a common consequence of lead vegetation [1,6]. *Staphylococcus aureus* remains a predominant pathogen in such cases, with methicillin-resistant strains contributing to worse outcomes [1,12].

Transvenous lead extraction is the cornerstone of managing device-related infections, achieving high success rates, and reducing mortality when combined with targeted antibiotic therapy [3,13]. However, the decision regarding device reimplantation remains contentious, particularly in pacemaker-dependent patients, as it increases the risk of reinfection with procedural risks of vascular injury and lead fragmentation [7]. The timing and site of reimplantation must be carefully evaluated to minimize reinfection risk, as shown in studies advocating delayed replantation in selected patients [6,7].

This case underscores the need for a multidisciplinary approach involving infectious disease specialists, cardiologists, and cardiothoracic surgeons. Such collaboration optimizes outcomes by ensuring timely diagnosis, effective surgical intervention, and tailored antibiotic therapy [8,13].

Advanced imaging modalities like transthoracic echocardiography (TTE) and pulmonary CT angiography played a pivotal role in diagnosing vegetation and thromboembolic complications in this case. These tools are increasingly recognized for their value in early detection and treatment planning [4,11].

The resolution of symptoms post-TLE and antibiotic therapy aligns with existing data that demonstrate significant clinical improvement with such interventions. Studies have also highlighted that patients with adequate follow-up imaging have better prognostic outcomes, emphasizing the importance of post-discharge monitoring [11,12].

While our case illustrates successful management, it also highlights gaps in understanding reinfection risk and optimal reimplantation timing. Further studies are required to standardize guidelines and improve outcomes for high-risk patients.

## Conclusions

This case highlights the pivotal role of TLE combined with prolonged antibiotic therapy in achieving favorable outcomes, aligning with current evidence from the literature. While the case demonstrates the effectiveness of early intervention, it underscores persistent gaps in optimizing reimplantation strategies such as optimal timing of lead extraction, site selection for reimplantation, alternative pacing options, and addressing reinfection risks. Further research is needed to establish standardized protocols for device reimplantation and evaluate the potential of innovative technologies, such as leadless devices, to mitigate infection risks. 
